# Handgrip strength and relationship with non-motor symptoms in Parkinson's disease

**DOI:** 10.1177/1877718X251340585

**Published:** 2026-05-20

**Authors:** Daniele Urso, Roberta Barone, Davide Vilella, Valentina Gnoni, Alessia Giugno, Lucia Batzu, Salvatore Minnella, Karolina Popławska-Domaszewicz, Kallol Ray Chaudhuri, Giancarlo Logroscino

**Affiliations:** 1 Center for Neurodegenerative Diseases and the Aging Brain, Department of Clinical Research in Neurology, University of Bari ‘Aldo Moro’, “Pia Fondazione Cardinale G. Panico”, Tricase, Lecce, Italy; 2 Department of Neurosciences, King's College London, Institute of Psychiatry, Psychology and Neuroscience, London, UK; 3 Principles and Practice of Clinical Research (PPCR), TH Chan Harvard School of Public Health, Boston, MA, USA; 4 Department of Neurology, Poznan University of Medical Sciences, Poznan, Poland; 5 Department of Translational Biomedicine and Neuroscience (DiBraiN), University of Bari 'Aldo Moro', Bari, Italy

**Keywords:** Parkinson's disease, sarcopenia, cognition, depression

## Abstract

**Background:**

Muscle weakness, particularly reduced handgrip strength (HGS), is a prominent clinical feature in Parkinson's disease (PD). While the relationship between motor symptoms and muscle strength has been extensively studied, the connection between HGS and non-motor symptoms (NMS) remains less explored.

**Objective:**

This study investigates the relationship between HGS and NMS in PD patients, hypothesizing that higher burden of NMS is associated with a reduced HGS.

**Methods:**

Fifty consecutive PD patients were enrolled and underwent comprehensive neurological and neuropsychological evaluations, 3T MRI scans, routine laboratory tests, and ^123^I-Ioflupane SPECT imaging. HGS was measured using a DynEx hand dynamometer, and NMS were assessed using the Non-Motor Symptoms Scale (NMSS). Cognitive function was evaluated with a battery of 18 psychometric tests, and quality of life was assessed using the Parkinson's Disease Questionnaire-8 (PDQ-8).

**Results:**

Reduced HGS was observed in the majority of PD patients and was significantly correlated with higher NMSS scores, particularly in mood and cognitive domains. Lower HGS was also linked to poorer quality of life. In linear regression models, NMSS remained a significant predictor of HGS even when controlling for age, sex, and Mini-Mental State Examination scores.

**Conclusions:**

Our findings suggest that HGS could serve as a valuable biomarker for non-motor symptoms, including depression and cognitive dysfunction, in PD patients. This study underscores the importance of integrating HGS measurements into clinical practice to identify muscle weakness and cognitive impairment, guiding more targeted interventions to improve patient outcomes.

## Plain language summary

This study looks at how handgrip strength is linked to non-motor symptoms in people with Parkinson's disease. Parkinson's disease is known for causing movement problems, but it also affects other areas like mood and thinking abilities. These non-motor symptoms can significantly impact a person's quality of life. We measured the handgrip strength of 50 people with Parkinson's disease and found that most had weaker grips than expected. We also assessed their non-motor symptoms using a scale that measures things like mood, attention, and memory. Our findings show that people with weaker handgrip strength often had more severe non-motor symptoms, especially mood and cognition. Additionally, those with weaker handgrips reported a lower quality of life. This suggests that handgrip strength might be a useful indicator of non-motor problems in people with Parkinson's disease. Doctors could use handgrip tests not only to check for muscle weakness but also to identify other issues like depression or memory problems, which could help improve treatment plans and patient outcomes.

## Introduction

Muscle weakness, particularly reduced handgrip strength (HGS), is a well-documented clinical feature in Parkinson's disease (PD) patients.^1^ Studies have consistently shown that individuals with PD exhibit lower HGS compared to healthy controls, highlighting the pervasive impact of the disease on muscular function.^1,2^ Recent meta-analyses have reinforced that both muscle strength and power are significantly impaired in PD,^3^ suggesting a strong correlation between reduced HGS and the severity of the disease.^4^ Reduced HGS can also define sarcopenia in these patients, a condition that is significantly more prevalent in PD than in the general population.^5,6^ Sarcopenia, characterized by the loss of muscle mass and strength, not only correlates with disease progression but is also associated with increased risks of falls and disability.^7–9^ The association between muscle strength deficits and PD is well-supported by epidemiological studies, which have demonstrated that a reduction in muscle strength, measured via handgrip strength, is linked to an increased risk of developing PD. One cohort study identified a significant relationship between low muscle strength at approximately 18 years of age and the subsequent incidence of PD later in life.^10^ Additionally, another study found that components of frailty, including slow gait speed and reduced grip strength, were associated with PD.^11^ These findings have been further corroborated by two recent studies that confirmed the association in older individuals across diverse populations.^12,13^ Despite these insights, whether muscle strength deficits constitute a true risk factor for PD or merely represent an early prodromal expression of the disease remains an area ripe for further investigation.

While the relationship between motor symptoms and muscular strength in PD has been extensively studied, the connection between HGS and non-motor symptoms (NMS) remains less explored. NMS in PD, which include cognitive impairments and mood disorders,^14^ are critical determinants of patients’ quality of life and often have a greater impact than motor symptoms.^15,16^ Evidence from studies in healthy elderly populations suggests that reduced muscular strength is associated with cognitive decline and depression.^17,18^ However, it is unclear whether these findings can be extrapolated to PD patients, where NMS are more prevalent and severe.

Given the significant burden of NMS on PD patients and the potential for HGS to reflect underlying pathophysiological changes, this study aims to investigate the relationship between HGS and NMS in PD. We hypothesize that reduced HGS is associated with a higher burden of NMS, particularly cognitive impairments and mood disorders. By exploring this relationship, we aim to provide insights into the role of muscular strength as a marker for NMS in PD, potentially guiding more targeted interventions to improve patient outcomes.

## Methods

### Participants

Fifty consecutive participants diagnosed with PD were enrolled in this study. These individuals were referred to the Center for Neurodegenerative Diseases and the Aging Brain at the University of Bari “Aldo Moro” (Bari, Italy) and the Pia Fondazione “Card. Panico” Hospital (Tricase, Italy) between January 2021 and January 2022. Each patient underwent a comprehensive assessment that included neurological and neuropsychological evaluations, a 3 T MRI scan, routine laboratory tests, and ^123^I-Ioflupane SPECT imaging as part of the diagnostic process. Demographic information, such as age and sex, was collected. The PD diagnosis was confirmed according to the latest Movement Disorders Society (MDS) criteria for clinically established PD,^19^ after excluding absolute exclusion criteria and red flags. All participants provided written informed consent, and the study was approved by the local ethics committee (ASL Lecce verbale No. 6, May 25, 2017), in compliance with the Declaration of Helsinki. Participants were selected solely based on clinical diagnosis, without any discrimination based on age, sex, or race.

### Clinical assessment

A comprehensive motor evaluation was performed using the MDS-Unified Parkinson's Disease Rating Scale motor part (UPDRS part-III). Disease stage was determined with the Hoehn and Yahr (H&Y) staging system. Baseline cognitive function was assessed with the Mini-Mental State Examination (MMSE). Disease duration was calculated from the date of the first motor symptom to the date of evaluation. The baseline burden of non-motor symptoms was assessed using the Non-Motor Symptoms Scale (NMSS). To better investigate the association between handgrip strength and individual non-motor symptoms domains, we also thoroughly examined the NMSS sub-domains individually. The Parkinson's Disease Questionnaire-8 (PDQ-8) was used to assess the quality of life. All evaluations, including neuropsychological and anthropometric assessments, were conducted in the “ OFF” medication state.

### Neuropsychological assessment

Each patient underwent a comprehensive neuropsychological evaluation, consisting of 18 psychometric tests, with at least two tests administered for each cognitive domain (memory, attention, executive function, visuospatial ability, and language). Raw scores from the cognitive tests were adjusted for age and education using published Italian normative data to calculate z-scores. The z-scores for each cognitive domain were summed, and mean group z-scores were calculated for each domain for every participant. For the memory domain, the Rey Auditory Verbal Learning Test (RAVLT) and the Rey-Osterrieth Complex Figure (ROCF – delayed recall) were administered. Executive function was assessed using the Digit Span Backward, Phonemic Fluency, Trail Making Test version B (TMT-B), and the Stroop Color-Word Test. Attention was evaluated with the Digit Span Forward, Trail Making Test version A (TMT-A), and the Symbol Digit Modalities Test (oral version). The visuospatial domain was assessed with the Italian Figure Copy Test,^20^ Rey-Osterrieth Complex Figure (ROCF), and the “Incomplete Letters” subtest of the Visual Object and Space Perception Battery (VOSP). The language domain was evaluated using the short version of the Boston Naming Test (BNT-15 items) and the verbal fluency test (both phonemic and semantic).

### Handgrip strength and anthropometric measures

Body composition was evaluated using bioelectrical impedance analysis (BIA) (BIA101 Anniversary, Akern s.r.l., Montacchiello, Italy). Anthropometric measures include weight, height, and body mass index (BMI) calculated taking a person's weight, in kilograms, divided by height, in meters squared. It provides an estimate of body fat in males and females of any age; the National Institute of Health (NIH) uses BMI to define a person as underweight, normal weight, overweight, or obese.^21^ The BIA was conducted in a controlled environment with temperatures between 24 and 26°C. Participants rested in a supine position for 15 min prior to the assessment. Electrodes were placed on the right hand and foot, and participants were asked to avoid alcohol consumption and strenuous exercise for 24 h before the test. The BIA metrics included BMI, skeletal muscle mass (SM, representing 40% of total body weight), appendicular skeletal muscle mass (ASM, the combined muscle mass of all four limbs), skeletal muscle index (SMI, calculated as SM divided by height squared), fat mass index (FMI, calculated as fat mass divided by height squared), and fat-free mass index (FFMI, calculated as fat-free mass divided by height squared). HGS was assessed using a DynEx hand dynamometer (Akern s.r.l., Montacchiello, Italy). Each hand's grip strength was measured twice, and the highest recorded value was used for analysis. According to the EWGSOP2 sarcopenia cut-off points, we classified handgrip strength as low if it was less than 27 kg for males and less than 16 kg for females.^22^ The MSRA-5 and SARCF-3 scores were also assessed as part of the sarcopenia evaluation. The MSRA-5 (Mini Sarcopenia Risk Assessment) is a self-reported questionnaire that includes five items assessing factors such as physical activity, history of falls, and weight loss, with a total score range of 0–60.^23^ Lower scores indicate a higher risk of sarcopenia. The SARCF-3 (Sarcopenia Questionnaire) is a simplified tool based on three key components: strength, assistance in walking, and chair rise, with scores ranging from 0–6.^24^ Higher scores indicate greater sarcopenia risk. Both tools were administered and scored according to their standard protocols. Physical activity levels were assessed using the International Physical Activity Questionnaire (IPAQ), which classifies individuals into low, moderate, or high activity levels.^25^ This was included to provide additional context for associations between activity levels, handgrip strength, and body composition.

### Statistical analysis

Data were explored using descriptive statistics (mean ± standard deviation or frequency). Normality was tested using the Shapiro-Wilk test. Statistical significance across groups (PD with high or low handgrip strength) was assessed using the chi-squared test, t-test, or Mann-Whitney U test, as appropriate. Correlations between HGS and demographic and anthropometric measures were determined using Spearman's rank correlation or Pearson correlation, depending on the data distribution. Similarly, correlations between HGS and clinical characteristics were established using partial Spearman's rank correlation or Pearson correlation, adjusted for age and sex. To explore the clinical predictors of HGS, we used univariable and multivariable linear regression models. Statistical analyses were performed using JASP 0.18.3 (https://jasp-stats.org/). The significance level was set at a p-value of 0.05.

## Results

Nineteen patients were categorized in the high HGS group, and thirty-one in the low HGS group. Demographic, clinical, and anthropometric characteristics are summarized in Table 1. The two groups did not differ in terms of age (p = 0.180), sex (p = 0.409), and education (p = 0.222). Clinically, patients with low HGS had lower scores on the MMSE (p = 0.036) and higher scores on the NMSS (p = 0.004), indicating a higher non-motor and cognitive burden. However, no differences were observed in disease duration (p = 0.622), MDS-UPDRS scores (p = 0.239), or Hoehn and Yahr stage (p = 0.444). Anthropometric evaluations showed no differences between the groups regarding BMI (p = 0.665), SM (p = 0.561), SMM (p = 0.242), FMI (p = 0.685), and FFMI (p = 0.882). However, MSRA-5 (p = 0.008) was significantly lower in the low HGS group, indicating an association between HGS and sarcopenia measures. Additionally, there was a trend toward higher SARCF-3 scores (p = 0.060) in the low HGS group. According to the IPAQ, the majority of patients were classified as having a low level of physical activity, with 46 (92%) patients falling into this category. Four patients (8%) were categorized as having a moderate level of physical activity, while no patients were classified as having a high level of physical activity.

**Table 1.** Demographic and clinical characteristics of Parkinson's disease with high and low HGS.

	PD - Total	PD with high HGS	PD with low HGS	Test Statistic (low vs. high)
n	50	19	31	
Age, y	68.46 (9.16)	65.42 (11.68)	70.32 (6.78)	W = 227, p = 0.180
Sex (male/female)	35/15	12/7	23/8	χ^2^ = 0.68, p = 0.409
Education, y	9.86 (4.05)	10.84 (4.50)	9.25 (3.69)	W = 353, p = 0.222
Disease Duration, y	4.79 (3.14)	4.42 (11.68)	5.10 (3.94)	W = 255, p = 0.622
MDS-UPDRS III	28.60 (11.86)	26.05 (10.98)	30.16 (12.28)	t = −1.19, p = 0.239
Hoeh and Yahr stage	1.92 (0.56)	1.84 (0.60)	1.96 (0.54)	W = 266, p = 0.444
MMSE	25.95 (4.13)	27.10 (3.69)	25.18 (4.30)	W = 362, p = **0.036**
NMSS total	53.95 (43.35)	31.61 (18.67)	67.36 (48.44)	W = 136, p = **0.004**
BMI	27.06 (4.42)	26.67 (4.80)	27.24 (4.27)	t = −0.43, p = 0.665
MSRA-5	51.72 (4.35)	53.94 (4.27)	50.35 (3.90)	W = 417, **p** **=** **0.008**
SARCF-3	1.46 (1.13)	1.05 (0.91)	1.71 (1.89)	W = 204, p = 0.060
SM	10.33 (1.70)	10.51 (1.67)	10.22 (1.75)	t = 0.59, p = 0.561
SMM	28.85 (6.35)	30.27 (5.79)	27.98 (6.60)	W = 353, p = 0.242
FMI	5.83 (3.61)	5.56 (3.52)	5.97 (3.72)	t = −0.41, p = 0.685
FFMI	21.15 (2.41)	21.08 (2.65)	21.19 (2.30)	t = −0.14, p = 0.882

Mean (standard deviation) scores are shown unless otherwise indicated. PD: Parkinson's disease; HGS: handgrip strength; MMSE: Mini-Mental State Examination; MDS-UPDRS: Movement Disorders Society Unified Parkinson's Disease Rating Scale; MSRA-5: Mini Sarcopenia Risk Assessment; SARCF-3: Sarcopenia Questionnaire; NMSS: Non-Motor Symptoms Scale; MMSE: Mini-Mental State Examination; BMI: body mass index; SM: skeletal muscle mass; SMI: Skeletal Muscle Index; FMI: fat mass; FFMI: Fat-Free Mass Index.

### Correlation of HGS with demographic and anthropometric measures

HGS correlated with sex (r = −0.445, p = 0.001) and age (r = −0.239, p = 0.048), indicating higher strength in males and decreasing strength with age. HGS also correlated with MSRA-5 (r = 0.499, p < 0.001) and SARCF-3 (r = −0.317, p = 0.025). Additionally, it was associated with SMI (r = 0.425, p = 0.002) and SMM (r = 0.563, p < 0.001), but not with FMI (r = −0.210, p = 0.144) or FFMI (r = 0.192, p = 0.181).

### Correlations of HGS with clinical variables

**Figure 1.**
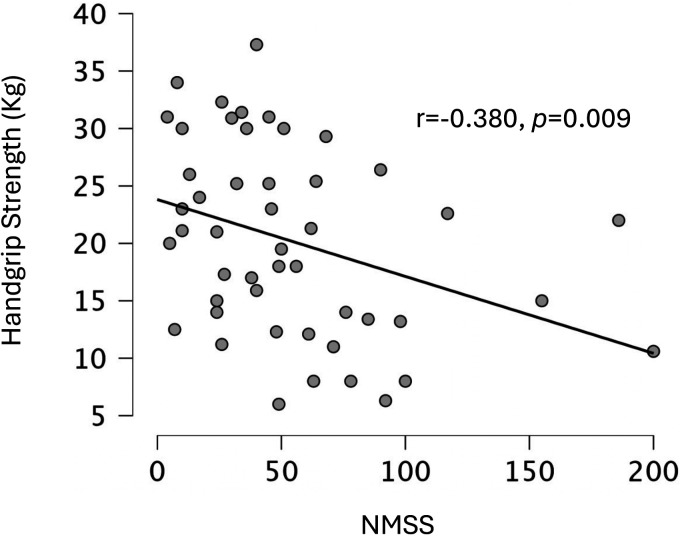
Associations between HGS (in kilograms) and scores on the non-motor symptoms scale (NMSS). Scatterplots showing the Spearman partial correlations (rs) after controlling for age and sex. The linear fit (solid line) is shown.

No significant correlations were found between HGS and Hoehn and Yahr stage (r = −0.107, p = 0.478) or motor severity as measured by MDS-UPDRS (r = −0.119, p = 0.430). However, HGS correlated with MMSE (r = 0.313, p = 0.041) and NMSS (r = −0.380, p = 0.009, Figure 1), indicating that lower HGS is associated with cognitive dysfunction and a higher burden of non-motor symptoms. Specifically, within the NMSS domains (Table 2), mood (r = 0.495, p < 0.001) and attention/memory (r = 0.336, p = 0.023) were significantly associated with HGS. These associations remained significant when analyzed separately by sex, except for the attention/memory domain in females. Among the five cognitive domains, attentional (r = 0.437, p = 0.002) and executive (r = 0.333, p = 0.021) domains were significantly positively correlated with HGS. Quality of life as measured by the PDQ was also significantly positively correlated with HGS (r = 0.393, p = 0.008).

**Table 2.** Spearman partial correlations between age HGS and NMSS in all patients and for male and female patients separately.

	All subjects	Male	Female
Cardiovascular	−0.273 (0.067)	−0.301 (0.095)	−0.279 (0.335)
Sleep/Fatigue	−0.183 (0.224)	−0.166 (0.363)	−0.124 (0.673)
Mood	**−0.495** (**<0.001)**	**−0.465** (**0.007)**	**−0.672** (**0.009)**
Perceptual Problems	−0.148 (0.325)	−0.187 (0.306)	−0.094 (0.749)
Attention/Memory	**−0.336** (**0.023)**	**−0.409** (**0.020)**	−0.207 (0.478)
Gastrointestinal	−0.286 (0.054)	−0.221 (0.225)	−0.487 (0.077)
Urinary	−0.113 (0.453)	−0.027 (0.885)	−0.371 (0.192)
Sexual Dysfunction	−0.049 (0.748)	−0.060 (0.745)	−0.136 (0.643)
**Miscellaneous**	**−0.333** (**0.024)**	−0.309 (0.085)	−0.512 (0.061)
Total	**−0.394** (**0.007)**	**−0.359** (**0.044)**	**−0.553** (**0.040)**

Correlation are indicated as r value (p value). Adjustments were done for sex and age in the overall group and only for age in the groups of male and females. The Non-Motor Symptoms Scale (NMSS) assesses non-motor symptoms across various domains: Cardiovascular (items 1–2), Sleep/Fatigue (items 3–7), Mood (items 8–12), Perceptual Problems (items 13–15), Attention/Memory (items 16–18), Gastrointestinal (items 19–22), Urinary (items 23–24), Sexual Dysfunction (items 25–26), and Miscellaneous (items 27–30).

### Clinical predictors of HGS

In the linear regression model (Table 3), NMSS was a predictor of HGS in both univariable (p = 0.015) and multivariable (p = 0.007) models, even when age, sex, and MMSE were included. In the multivariable model, MMSE lost its significance as a predictor.

**Table 3.** Clinical predictors of HGS in Parkinson's disease.

	Univariable analysis		Multivariable Analysis	
Variables	Estimate (95% CI)	*p*	Estimate (95% CI)	*p*
Age	−0.22 (−0.48, 0.04)	0.094	−0.05 (−0.314, 0.20)	0.659
Sex	−8.13 (−12.87, −0.38)	**0**.**001**	−7.93 (−12.39, −3.47)	**<0**.**001**
NMSS	−0.07 (−0.12, −0.01)	**0**.**015**	−0.08 (−0.14, −0.02)	**0**.**007**
MMSE	0.64 (0.05, 1.24)	**0**.**047**	0.13 (−0.47, 0.72)	0.675

Estimate is unstandardized coefficient.

NMSS: Non-Motor Symptoms Scale; MMSE: Mini-Mental State Examination,

## Discussion

In this study, we observed that the majority of patients with PD exhibit reduced HGS and that this reduction correlates with the burden of non-motor symptoms in both males and females. Specifically, our findings show that among the non-motor symptoms, mood-related symptoms and cognitive domains, particularly attention, are most strongly associated with low HGS. Furthermore, we observed that reduced HGS is linked to a lower quality of life.

The association between reduced muscle strength and non-motor symptoms in PD on the one hand aligns with existing literature that identifies non-motor symptoms as crucial determinants of quality of life and critical factors in the overall progression and severity of PD.^14^ Notably, non-motor symptoms significantly impact disability^26^ and mortality,^27^ serving as indicators of a more malignant disease. This underscores the concept that non-motor symptoms are linked to a poorer overall and disease-specific prognosis. Our findings are consistent with studies in the general population, which have demonstrated that HGS is associated with cognitive impairment and depression.^17,18^ Several cross-sectional and longitudinal studies have shown that measures of HGS are linked to cognitive declines regardless of age demographics and the presence of comorbidities^17^ Similarly, a recent large-scale study involving 115,601 participants followed for more than three years found an inverse significant association between each kilogram increase in HGS and depression. This suggests that muscular strength may serve as a preventive factor for depression in older adults.^18^ Therefore, our findings indicate that HGS could be a valuable biomarker for both non-motor symptoms and cognitive function in PD patients.

Particularly interesting is the relationship between muscular strength and cognitive functions, especially attention, which is a higher cognitive function involved in response selection and inhibition through close interactions with the motor system.^28^ Recently, two studies have investigated these relationships using various methodologies.^29,30^ Rinne et al.^29^ tested whether influences of attention control are also seen on lower-level motor functions of dexterity and strength by examining relationships between attention control and motor performance in healthy-aged and hemiparetic-stroke subjects. They found that dexterity and force generation require intact attention control.^29^ Likewise, Chong and colleagues^30^ examined these relationships in 148 community-dwelling older adults, finding that higher HGS was related to better processing speed, attention, and global cognition. Using functional MRI, they also found that higher HGS was associated with greater segregation of the salience/ventral attention network.^30^ These findings are particularly intriguing as they suggest that the association between strength and cognition is observed not only in healthy older adults but also in individuals with neurodegenerative diseases. This highlights the need for further research to unravel the underlying mechanisms linking muscular strength to cognitive functions and to explore potential therapeutic implications.

Another significant finding is the association between muscular strength and quality of life. Although this has not been extensively studied in PD populations, there is substantial literature on this association in other pathologies^31,32^ and older adults.^33,34^ Therefore, our finding may suggest that HGS may be a simple and practical indicator of quality of life in PD.

Previous literature indicates that the proportion of reduced HGS and sarcopenia is higher in the PD population compared to the general population, which is consistent with our findings of a high proportion of subjects with reduced hand strength.^1,2^ Recent meta-analyses have reinforced that both muscle strength and power are significantly impaired in PD.^3^ Furthermore, similar to our study, another study found that HGS predicted motor impairment only in a subgroup and not in the overall patient group.^4^ A high proportion of sarcopenia in PD patients has been identified regardless of the diagnostic criteria used, though there are some differences depending on the criteria applied.^6^ In our sample, HGS correlated well with MSRA-5 but less so with SARCF-3, highlighting how different measures of sarcopenia may agree to varying degrees.

Although our cross-sectional study cannot disentangle causal relationships between muscular strength and non-motor symptoms, we can cautiously hypothesize that these relationship between muscular strength and non-motor symptoms are bidirectional. Previous cross-sectional studies have reported that patients with major depressive disorder and cognitive disorders, including dementia, exhibit reduced HGS.^35^ Furthermore, longitudinal studies indicate that muscle strength loss often precedes the onset of cognitive decline and depression.^36,37^ Interestingly, randomized controlled trials have shown that strength training can improve depression^38^ and cognitive function.^39,40^ For example, a study found that twelve months of once-weekly or twice-weekly resistance training benefited the executive cognitive function of selective attention and conflict resolution among senior women.^39^ Current evidence from systematic reviews suggests that physical exercises, including strength training, positively impact both motor and non-motor outcomes in individuals with PD.^41^ Moreover, recent Mendelian randomization studies have shed light on the directionality of these relationships. While the association between depression and muscular strength appears unidirectional, cognitive impairments may exhibit a more complex bidirectional relationship.^42,43^ Despite the inherent limitations of Mendelian analyses, these findings underscore the potential of strength-based interventions in mitigating non-motor symptoms in PD. However, further longitudinal and mechanistic studies are required to confirm these hypotheses and explore underlying pathways.

The main limitation of our study is the lack of a control group. However, the primary aim was to explore the relationship between non-motor symptoms and hand strength rather than differences with controls, which are well-documented in the literature.^3^ Another limitation is the cross-sectional design, which precludes us from making causal inferences. Additionally, the majority of patients in our sample were classified as having low physical activity levels according to the IPAQ, with only a small subset showing moderate activity and none classified as highly active. While this makes the patient group more homogeneous, it limits the generalizability of our findings to individuals with higher levels of physical activity. Future studies should aim to elucidate the direction of causation and potential mediators in this association, which could aid healthcare providers and interventionists in preventing weakness, depression, and cognitive impairment. Despite these limitations, our study has several strengths. Notably, we analyzed associations stratified by sex, providing a more nuanced understanding of these relationships. Additionally, we validated our measure of strength by including various anthropometric measures, such as sarcopenia scales and bioimpedance analysis. The fact that HGS correlated with skeletal muscle mass but not with fat mass further supports its role as a biomarker for muscle health in PD patients.

HGS could potentially serve as a valuable marker indicating the burden of non-motor symptoms, particularly depression and cognitive dysfunction, in PD patients. Incorporating measures of HGS into clinical and epidemiological settings could help identify not only muscle weakness but also cognitive impairment. These insights could guide more targeted interventions aimed at improving both motor and non-motor outcomes, ultimately enhancing the quality of life for individuals with PD.
